# Research on the Spatial Correlation of China’s Haze Pollution and the Government’s Cooperative Governance Competitive Strategy

**DOI:** 10.3390/ijerph20010013

**Published:** 2022-12-20

**Authors:** Shijin Wang, Guirong Ji, Zhaolian Hu, Fangdao Qiu

**Affiliations:** 1 School of Business, Jiangsu Normal University, Xuzhou 221116, China; 2 School of Geography, Geomatics and Planning, Jiangsu Normal University, Xuzhou 221116, China

**Keywords:** cooperative governance, analysis of social networks, two-district system, race to the bottom, competitive strategy

## Abstract

A government’s choice of environmental strategy plays an important role in the coordinated governance of regional air pollution. Based on changes in China’s environmental policies and on changes in environmental indicators over the years, this paper selects regional haze data from the years 2005, 2009, 2013, and 2017; uses social network analysis to describe the structural characteristics of a spatial correlation network in China; measures the level of coordination using a population gravity model; and further analyzes the influence of the overall structural characteristics of spatial networks on the level of coordination. The results show that the spatial association of regional haze presents a typical “central edge” network structure. The Beijing–Tianjin–Hebei region and the Yangtze River Delta region are the largest emitters in China. The coordination level of haze control in China showed a fluctuating upward trend, but the overall level of coordination is relatively low, and there is still great room for improvement. Based on the above characteristics, using the provincial panel data from 2005–2017, a two-zone spatial Durbin model was built to empirically test the impact of changes to the environmental performance assessment system on local coordinated haze-control decisions and their stage characteristics. The overall sample results show that there was a “race to the bottom” among Chinese provinces during the study period. When the haze control intensity in neighboring areas was relaxed, the regional governments also tended to relax their own environmental regulation intensity. The time-based analysis results further show that with the improvement of the environmental performance assessment system, the strategy selection of coordinated governmental haze-management presents the possibility of a “race to the top”.

## 1. Introduction

Since the end of the last century, both the central Chinese government and the local Chinese government at all levels have introduced a series of environmental regulations, policies, and measures—including the “low-carbon economy” and “beautiful China” policies; the treatment of pollution prevention and control as one of the three major battles to build a well-off society; and the building of a modern environmental governance system—with the aim of promoting the reconstruction of the environmental regulation system, as well as controlling the production of environmental pollution. In terms of the environmental assessment index system, in 2007, environmental constraint indicators were officially included in the assessment systems at all levels of government in China, and the “ecological assessment” system was formally implemented. With the severe air quality problem, the central government began to introduce a “one-vote veto system” in 2011. After 2015, China began to implement an environmental supervision mechanism and an accountability system that allowed leading officials to be held accountable for environmental damage that occurred under their supervision. On the other hand, the official ecological assessment index system, which started in that year, included SO_2_ emission-reduction indicators without CO_2_ constraints (not used as a mandatory indicator of official assessment until 2014), thus creating conditions for observing the sensitivity of local government pollution-emission-reduction performance to the settings of official assessment indicators. Since 2019, the Ministry of Ecology and Environment has released a number of air control plans, such as the 2018 China Ecological Environment Bulletin, the Comprehensive Treatment Plan for Volatile Organic Compounds in Key Industries, the Comprehensive Control Plan for Air Pollution in Industrial Kilns, and the Guiding Opinions on Coordinating and Strengthening Work Related to Coping with Climate Change and Ecological and Environmental Protection. While promoting control of air pollution, it is also necessary to coordinate the control of greenhouse gas emissions and to promote the synergy of climate change and ecological and environmental governance. In November 2019, all 12 transmission channels for air pollution prevention and control were completed and put into operation. Since the introduction of the environmental protection tax, the tax governance capacity and environmental governance capabilities have been “doubly improved”, effectively helping to actively win the battle against pollution and actively combat climate change. In 2019, national environmental protection taxes reached CNY 22.117 billion, of which CNY 19.711 billion was taxed, accounting for 89.1%. At the same time, the environmental protection tax gives two tax cuts for low-standard emissions, in order to encourage enterprises to update environmental protection equipment, improve technology, reduce pollutant emissions, and reduce the emissions of major air pollutants. In recent years, although environmental performance has achieved a certain degree of success, environmental governance and ecological performance assessment still need to be improved, especially with respect to regional government competition strategies and measures in the process of climate governance.

## 2. Literature Review

The effects of implementing a regional environmental cooperation/competition strategy shows that the effects of multilateral collaborative control are better than those of bilateral or unilateral control, and that environmental policies with incentive effects are conducive to promoting the development of multilateral cooperation, while the negative impact of hitchhiking behavior should be prevented to ensure the fairness of collaborative cooperation. Porter [1] believes that countries with rapid industrialization have the most “bottom-by-bottom competition”, because the high environmental standards of foreign trade will reduce their value and reduce their own level of environmental protection regulation. Woods [2] used data from the United States to show that, because of the inconsistency in environmental regulation levels, states with low environmental regulation levels will “maintain the status quo”, while states with high environmental regulation levels will “look to the low”, pursue economic interests, and constantly reduce their environmental performance appraisal targets. Renard [3] also carried out “bottom-by-bottom competition” asymmetric reaction experiments on China’s six-year data. The study found that local environmental regulatory strategies will interact in various regions of China due to resource acquisition, but that the phenomenon of “bottom-by-bottom competition” is not frequent. Tacconi [4] believes that to promote regional cooperation in haze control in southeast Asia, environmental cooperation agreements should be signed to formulate effective incentive and punishment measures, with developed regions in particular being the focus of governance costs. Paterson [5] analyzed the huge amount of pollution caused to the national environment by the South African mining industry; they concluded that governmental management lacked the ability to maintain natural resources and to protect the ecological environment, and proposed that cross-regional collaborative governance is necessary to enhance governmental control of large enterprises and to improve environmental quality. Forsyth [6] analyzed the effects on southeast Asian residents of forest fires in Indonesia, Singapore, and Malaysia caused by cross-border haze pollution. Local residents responded by scolding, appealing, and marching to promote government-related environmental protection policies. In response to these kinds of public concerns about cross-border haze pollution, southeast Asian countries should establish a cross-border haze agreement, in order to prevent regional haze pollution from continuing to increase. Kim [7] analyzed the events related to South Korea’s participation in comprehensive regional environmental governance, discussed the mechanism of northeast Asia, and believed that South Korea has played a huge role in leading environmental cooperation in northeast Asia, but that regional environmental cooperation in northeast Asia still faces great challenges. Lee [8] studied the Japanese government’s efforts with respect to haze pollution control in recent years, and found that the Japanese government’s territorial governance is very limited with respect to environmental governance. It was found that outside haze pollution is an important cause of Japanese environmental governance failure, and the author further emphasized the necessity of Japan and South Korea collaborating to control haze. Scrimali and Mirabella [9], through game theoretic analysis of pollution reduction in multiple countries, explored the key factors affecting emission reduction and the establishment of a collaborative emission-reduction framework. Omidvarborna et al. [10] studied the available regulations, emissions lists, and sources of pollution in GCC member-states to facilitate air-pollution assessment and coordinated air-pollution governance in these developing countries. At present, the research on collaborative control of regional haze focuses on discussing the necessity of cooperation, system design, and countermeasures and suggestions, and focuses less on the overall evaluation of the level of regional haze coordination, how to form a coordinated mode of haze control, and how to make such control sustainable and stable. Yang [11] verified the interaction between economic growth and environmental regulation based on spatial correlations in China. The study found that the comparison/competition phenomenon is relatively common, and that blind following has become the mainstream. Zhang et al. [12] used the two-district model, considering both accounting and explanatory variables, to verify the existence of strong “bottom-by-bottom competition” in regional environmental regulation in China. When performance assessments take GDP as the main investigation target, local governments pursue fiscal goals and relax the environmental assessment goals. Li [13] used a dynamic game model to demonstrate the “bottom-by-bottom competition” phenomenon among Chinese regional governments under the goal of land finance. Zhao et al. [14] analyzed the possibility of “bottom-by-bottom competition” from the FDI perspective of 286 prefecture-level cities in China, which were further verified. Liu [15] notes that the central government has included environmental constraint indicators in the assessment system for governments at all levels since 2007, but argues that the phenomenon of “bottom-by-bottom competition” is still common in this ecological assessment because of the strategic intergovernmental competition between China local governments.

## 3. Data Analysis

Social network analysis is based on the associations between different subjects, from the perspective of relationship data; it comprehensively weighs the relationship between population flow and flow direction between different regions, and thus describes the dynamic change process of group agglomeration and contact relationships in population migration, in to make up for the defects in the original attribute data.

### 3.1. Population Gravity Model

The population gravity model is used to construct the data system needed for the analysis of haze pollution networks in various regions [16]. Gravitation models can be used to measure interregional population migration, in the initial form of:

(1) $$M_{ij}=K\frac{P_{i}P_{j}}{D_{ij}},$$

In Formula (1), $M_{ij}$ indicates the total size of the migratory population from region *i* to region *j*; K indicates the constant; $P_{i},P_{j}$ indicate the populations regions *i* and *j*, respectively; and $D_{ij}$ indicates the distance between region *i* and region *j*.

As population migration gradually increases due to economic factors, the corresponding population agglomeration area appears to develop a haze pollution problem. [17] Therefore, this paper aims to analyze these patterns using the gravity model, in order to consider the influence of economic factors and geographical distance on haze pollution control, and to form a final model. The specific formula to be used is the following:

(2) $$T_{ij}=K\frac{\sqrt{P_{i}\times G_{i}}\times \sqrt{P_{j}\times G_{j}}}{D_{ij}^{b}},\;\left(K=\frac{G_{i}}{G_{i}+G_{j}},i\;\neq \;j,i=1,\;2,\;3\cdots n,j=1,\;2,\;3\cdots n\right)$$

where $T_{ij}$ indicates the intensity of haze pollution between provinces *i* and *j*, $P_{i}\;\mathrm{and}\;P_{j}$ indicate the levels of haze pollution in provinces *i* and *j* in that year,$\;G_{i}\;\mathrm{and}\;G_{j}$ indicate the GDP levels for the corresponding provinces, $D_{ij}$ indicates the shortest transportation distance between provinces *i* and *j*, and *b* indicates the distance decay index (since haze is characterized by a two-dimensional variable, *b* is taken to be 2 here).

### 3.2. Spatial Correlation Effect of Haze Pollution in Various Regions in China

This paper analyzes environmental assessment data from 30 provincial administrative units from 2005–2017 to provide empirical evidence of cross-regional correlations between regions, based on geographical law (that is, terrain characteristics), resource endowment and humanistic characteristics, and the policies of local governments to develop their local economies (especially the system of scale competition and promotion championship).

**Figure 1:**
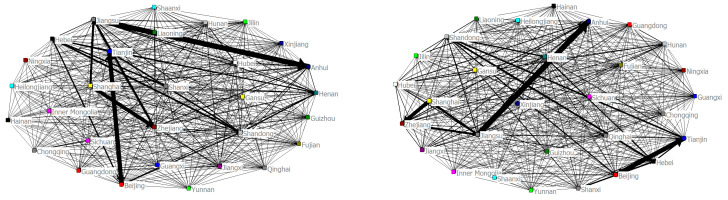

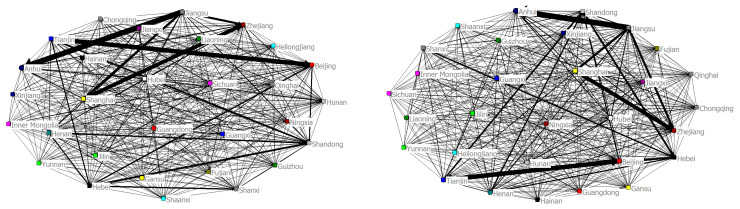
Haze-pollution-related networks in various regions in 2005, 2009, 2013, and 2017.

As can be seen from Figure 1, in 2005, there were major associations between haze pollution levels in the provinces of Jiangsu and Anhui, Shanghai and Zhejiang, and Shandong and Hebei, indicating that there are strong haze pollution associations between the above six provinces, and that the spatial connection between areas of haze pollution is high. Additionally, Zhejiang was more haze-pollution-associated with Jiangsu, Shanxi with Hebei, Beijing with Tianjin, and Shandong with Henan, as compared with other provinces. Haze pollution has a strong regional correlation, especially in the Beijing–Tianjin–Hebei urban agglomeration and the Yangtze River Delta urban agglomeration.

In 2009, Jiangsu and Anhui, Shanghai and Zhejiang, and Shandong and Hebei had a strong haze pollution correlation, but the main difference from 2005 was that the association between haze pollution levels in Shanghai and Jiangsu reduced, while the haze pollution correlation in the Beijing–Tianjin–Hebei region still showed a large correlation.

In 2013, the association between haze pollution levels in major provinces was greatly different from that in 2009, mainly manifested in the intensity of haze pollution association between Shanghai and Zhejiang. The main haze pollution association in China at the time existed between Anhui and Jiangsu, and Shandong and Hebei. At the same time, the intensity of haze pollution association between Shanghai and Jiangsu further decreased from 2009. The provinces of Shanghai and Jiangsu, and Shanghai and Zhejiang made great progress in haze pollution control. At the end of 2013, a haze swept through most provinces and cities in China, and lasted for several months. The haze phenomenon in the Yangtze River Delta region in particular was relatively serious, and had a great negative impact on people’s health and their normal production and life. In early November 2013, the air quality in the Yangtze River Delta region began to decline, and with the continuous decline of air quality, the air visibility got lower and lower, even affecting people’s ability to travel. The warning for air quality once rose from yellow to red. Shanghai and Nanjing in particular, among other areas, had the worst air pollution, with a red warning. The concentration of PM_2.5_ soared, exceeding about ten times the national standard. Locals said the smog was the worst in the Yangtze River Delta region. In Nanjing, Jiangsu Province, PM_2.5_ once reached an instantaneous concentration of 943 micrograms, forcing schools in some areas to stop holding classes. Therefore, it is necessary to investigate the causes of haze in the Yangtze River Delta region. On September 10, 2013, the Prevention and Control of Air Pollution Action Plan was issued, which has been called the strictest environmental policy in the history of New China. The policy focuses on the Beijing–Tianjin–Hebei region and its surroundings, the Yangtze River Delta region, and the Pearl River Delta region, and has deployed a series of measures such as comprehensive governance, industrial structure, energy structure, and regional cooperation, and clarified the goal of reducing PM_2.5_ by 2017. 

As can be seen from the figure for 2017, great results were achieved in haze pollution control compared with 2013. The haze pollution correlation between Anhui and Jiangsu and between Hebei and Shandong was reduced, while the haze pollution correlation between other provinces was small, indicating that in the four years since 2013, major provinces in the country had made effective achievements in haze pollution control. According to an assessment by the Ministry of Ecology and Environment in May 2018 and the National Development and Reform Commission, from 2013–2017, the three key regions of Beijing–Tianjin–Hebei, Yangtze River Delta, and Pearl River Delta achieved remarkable results in reducing the average PM_2.5_ concentration to 39.6%, 34.3%, and 27.7% respectively. However, the environmental pollution problem represented by “haze” still exists, and the prominent contradiction between economic development and environmental quality has not been fundamentally changed. Urban agglomerations are a major engine to promote social and economic development in the region. In the process of traditional extensive urbanization, air pollution problems caused by excessive population agglomeration, traffic jams, and excessive energy consumption are gradually exposed. In particular, the contradiction between urbanization, on the one hand, and the protection of resources and the environment, on the other, is especially acute and prominent in China’s economically developed eastern urban agglomeration, which is the primary agglomeration area of haze pollution. In 2017, the urbanization rates of Shanghai, Jiangsu, Zhejiang, and Anhui in the Yangtze River Delta were as high as 87.7, 68.76, 68.00, and 53.50%, respectively. This rapid urbanization inevitably brought great pressure and challenges to the local ecological environment, especially the air quality.

Through the above social network relationship analysis, we can determine the structural characteristics of the spatial association network of regional haze emissions in China, and effectively prove that haze pollution has a strong spatial correlation effect in China, thus explaining the necessity of coordinated haze control. At the same time, based on the population gravity model, this paper proves that the degree of coordination with respect to haze control in China is at a relatively low level, which reflects the urgency of coordinated haze control. Further sub-regional analysis reveals that the focus of haze management should be in the surrounding areas of the Beijing–Tianjin–Hebei region and the Yangtze River Delta region.

## 4. Research and Design

From the four key nodes of 2005, 2009, 2013, and 2017, China presents strong stage characteristics in regional air pollution control. From the perspectives of top-level design and practical strategy, the mechanism of joint prevention and control of air pollution should be explored and gradually established to provide strong support for comprehensively winning the "battle of protecting the blue sky”. From a top-level design perspective, China’s joint prevention and control of air pollution can be roughly divided into four stages: the institutional exploration stage (1988–2007), the preliminary proposal stage (2008–2010), the stage of development enrichment (2011–2014), and the deepening and expansion stage (2015 to the present). In the stage of institutional exploration, the joint prevention and control of air pollution began to take shape. In 1988, to solve the problem of compound air pollution, the State Council approved the “Five-Year Plan” for the Prevention and Control of Acid Rain and Sulfur Dioxide Pollution in the Two Control Areas, and proposed the establishment of “two control zones” for acid rain and sulfur dioxide; this was essentially the prototype for the joint prevention and control of air pollution. In the 11th Five-Year Plan proposed in 2005, it was suggested for the first time to reduce energy consumption per unit of GDP, and to clarify the binding indicators for the discharge of major pollutants [18]. In 2007, as the content of environmental constraints indicates, the government performance assessment saw the first “inflection point” in environmental governance, and sulfur dioxide emissions and chemical oxygen demand decreased for the first time. In 2010, the State Council issued the Guiding Opinions on Promoting the Joint Prevention and Control of Air Pollution and put forward the concept of “regional joint prevention and control of regional air pollution” for the first time; this proposed the division of coordinated control areas, and established a working mechanism for regional joint prevention and control. In the stage of rich development, the control method combining total control with quality was established for the first time. In 2012, the 12th Five-Year Plan for the Prevention and Control of Air Pollution in Key Areas was issued by the Ministry of Environmental Protection. It divided the country into coordinated governance areas according to the characteristics and flow laws of air pollution, and proposed the establishment of a regional coordinated control mechanism as well as a joint law enforcement and supervision mechanism in key areas. Since then, the State Council has successively issued the 13th Five-Year Plan for Ecological and Environmental Protection as well as the Three-Year Action Plan for Winning the War to Protect the Blue Skies, and the Ministry of Environmental Protection has issued the Key Points for the National Prevention and Control of Air Pollution. So far, China’s regional joint prevention and control mechanism for air pollution has been deepened, refined, and implemented, and different joint prevention and control entities have comprehensively promoted the regional joint prevention and control of air pollution.

At the practical strategy level, China has taken a series of strict clean air actions. In 2019, 157 of 337 cities reached the ambient air quality, up 58.59% over 2017 (338 cities). The average proportion of good days in 337 cities reached 82.0%, 4 percentage points higher than the 78% in 2017. The PM_2.5_ concentration of 36 μg/m^3^, decreased by 16.28% from 2017. To study whether the changes in the governmental performance appraisal system can bring about the upgrading of local collaborative haze control decisions, we will divide the data into four stages based on the year: the first stage is from 2005–2007, the second from 2008–2010, the third from 2011–2014, and the fourth from 2015–2017.

### 4.1. Model Setting

Due to the differences in environmental regulations and haze concentration across regions, the intensity and speed of regulatory responses can also vary across neighboring regions in different provinces. To study the collaborative governance regarding haze control under conditions of “government heterogeneity”, the two-zone spatial Durbin model is introduced for analysis.

### 4.2. Space Autocorrelation Test

In performing the spatial measurement analysis, the general features of the correlation between variables are measured by the global *Moran’s I* index, formulated as follows:

(3) $$Moran’s\;I=\frac{\sum_{i=1}^{n}\sum_{j=1}^{n}w_{ij}\left(x_{i}-\bar{x}\right)\left(x_{j}-\bar{x}\right)}{S^{2}\sum_{i=1}^{n}\sum_{j=1}^{n}w_{ij}}$$

Local autocorrelation analysis can be used to detect the spatial correlations that the samples exhibit in local spatial subsystems, and the local *Moran’s I* index can be used to reflect individual agglomeration features, expressed as follows:

(4) $$Mroan’s\;I=\frac{\left(x_{i}-\bar{x}\right)\sum_{i=1}^{n}\sum_{j=1}^{n}W_{ij}{\left(x_{i}-x_{j}\right)}^{2}}{S^{2}}$$

$w_{ij}$ is an element in the spatial weight matrix w located in i rows and j columns. The weight matrix is determined by the spatial adjacency relationship. If region i is adjacent to region j, $w_{ij}$ is 1, and if not, it is 0. $x_{i}$ and $x_{j}$ represent haze concentrations in two regions, i and j. n is the number of regions. The value of the local spatial autocorrelation for *Moran’s I* exponent is [−1, 1].

### 4.3. Two-Zone System of Space Model

In the general measurement analysis, the variables are assumed to be independent, while the spatial measurement analysis considers the spatial relationships between regional geographical objects, revealing the characteristics of spatial dependence and spatial correlation. Spatial metrology models introduce the mutual influence of geographic space to modify the classical regression model by establishing a spatial weight matrix to obtain efficient estimation coefficients. [19] However, the traditional spatial linear model cannot measure the asymmetric effects under different regions. Based on this, this paper uses the two-zone spatial model proposed by Elhorst [20] to set different spatial correlation coefficients for different regulatory forces and to test the asymmetric effects. Compared to the spatial error model and the spatial lag model, the spatial Durbin model introduces the spatial lag explanatory variable, which is more applicable in the strategy interaction model. Therefore, we set a two-zone spatial Durbin model:

(5) $$Y_{it}=\rho_{1}d_{it}\sum_{j=1}^{30}w_{ij}Y_{jt}+\rho_{2}(1-d_{it})\sum_{j=1}^{30}w_{ij}Y_{jt}+\beta X_{it}+\theta \sum_{j=1}^{30}w_{ij}X_{jt}+\alpha +\mu_{it}+\lambda_{t}+\varepsilon_{it}$$

(6) $$d_{it}=\left\{\begin{array}{l} 1,Y_{it}\;>\;\sum_{i=1}^{30}w_{ij}Y_{it},\;i\neq j\;\\ 0,other \end{array}\right.$$

where $Y_{it}$ indicates the phase t regulation intensity of province i; $X_{it}$ is the control variable; slope $\rho_{1}$ measures the response strength of province i more than competing provinces; slope $\rho_{2}$ measures the response intensity of province i less than competing provinces; β is the influence of the regional control variable on the strength of regulation; θ is the influence of the control variable in the neighboring region; α is the intercept term; $\mu_{it}$ indicates individual fixed effect; $\lambda_{it}$ indicates time-fixed effect; and $\varepsilon_{it}$ is the error term.

The indicator variable $d_{it}$ in the form of 0–1 virtual variable reflects the dynamic game characteristics of the competition intensity of each province under environmental regulation. Measured by Formula (6), the variable equals 1 when the regulation intensity is greater than that of the competing province and 0 when the regulation intensity is less than in the competing province. Therefore, the state evolution of the strategy under “government heterogeneity” can be determined by judging the magnitude of the environmental regulatory term reaction coefficients, $\rho_{1}$ and $\rho_{2}$, under the spatial game model. “Bottom-by-bottom competition” is a kind of “relaxed” strategy choice in the environmental regulation competition. That is, when a rival province relaxes its environmental regulation levels, local governments tend to imitate its behavior more strongly than if the rival province had made its regulations more strict; this is reflected by the variables $\rho_{1}$ > $\rho_{2}$. Conversely, a “top-by-top competition” refers to a “strict” environmental regulation competition strategy choice, reflected by $\rho_{1}$ < $\rho_{2}$; in this case, local governments are more likely to imitate rival province behavior when the rival makes its environmental regulations more strict.

### 4.4. Variable Selection and Data Description

(1) Regulatory intensity (*RS*). Due to the challenging nature of trying to measure environmental regulation, scholars generally choose some alternative indicators to indirectly represent regulation intensity. At present, environmental regulation is mainly measured by three indicators: one is to represent the effectiveness of policy implementation with specific measures that reflect the strength of environmental regulation; second is to establish environmental regulation levels based on the perspective of input and output; and third is to establish a comprehensive index system of pollutant discharge to measure the intensity of pollutant discharge. Several specific commonly used methods are pollutant discharge fee, pollution control investment per unit output value, comprehensive pollutant indicators, and pollution intensity [21,22,23]. For the present study, we follow the practice of Zhang (2010), who measures regulation using the ratio of total investment projects between industrial added value and industrial pollution control. The essence of the investment in pollution control is to reduce the pollution displacement. The greater the regulation, the smaller the discharge of the province is likely to be for the unit industrial added value, and so the stricter the environmental control of the region.
(2) Haze concentration (*HC*). When regional haze pollution is more serious, the local government will go to great efforts to control the haze. However, for the spatial diffusion characteristics of haze, the government is more inclined to relax environmental regulations. Because haze data is not directly available, proxy variables are needed to represent them. Considering that PM_2.5_ is the main component of haze, this paper draws the practice of Wang [24] to use the average annual PM_2.5_ concentration to characterize the level of haze pollution.
(3) Other control variables. ① Per Capita Gross Domestic Product (*PG*). The regulatory ability of local governments to deal with environmental regulation is related to the economic level, and the level of economic development determines the size of the potential market demand. The less developed the province, the more incentive there is to attract investment by lowering the regulatory threshold [25]. Therefore, the per capita GDP of various provinces and cities is used as a proxy variable for the level of economic development. ② Population Density (*PD*). Population density can reflect the size of regional competition pressure and the health of competition mode. Large cities with high population density pay more attention to sustainable environmental development and more strict regulation levels. Therefore, this paper measures the population level with the average annual population of provinces and cities. ③ Energy Strength (*ES*). Energy strength reflects the intensity of pollution in the area, and there are more pollution enterprises in areas with high energy intensity. Therefore, such provinces are more inclined to use access thresholds and environmental regulations as policy tools to compete with the economies of neighboring areas. In this paper, the effect of energy intensity is tested using the ratio of energy consumption to GDP. ④ Level of Urbanization (*URB*). Areas with a high level of urbanization pay more attention to sustainable economic development and have less pressure to attract capital entry. Therefore, they do less to participate in the vicious competition of “competition to the end”. Therefore, the ratio of urban population to total population is used as an indirect index reflecting the level of urbanization. ⑤ Industrial Structure (*IND*). The economic attributes of industry are closely related to environmental regulation, so the ratio of secondary industry GDP to regional GDP is taken as the proxy variable for secondary industry.

In this paper, data samples are taken from 30 provinces and cities (excluding Tibet, Hong Kong, Macao, and Taiwan) from 2005 to 2017, and the missing values in individual years were filled in using the interpolation method. Among these, raw data regarding PM_2.5_ were obtained for analysis from raster data from the Atmospheric Component Analysis Group at Dalhousie University. In addition to the haze concentration statistics, the original data used are taken from the China Statistical Yearbook, China Energy Statistical Yearbook, China Environmental Yearbook, and the Statistical Yearbooks of various provinces.

## 5. Empirical Results and Analysis

### 5.1. Spatial Correlation Analysis

**Figure 2:**
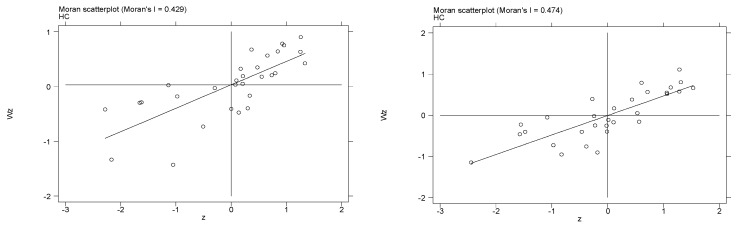
Local *Moran’s I* scatter plot of haze concentration in 2005 and 2017.

In this paper, the spatial correlation of haze pollution was tested based on *Moran’s I* index, using the Geoda software. Table 1 shows the results of global autocorrelation analysis tests for interprovincial PM_2.5_ concentrations in China from 2005–2017. The *Moran’s I* index of the haze concentration during the sample investigation period was positive and passed the significance level test of 1%, indicating that the haze pollution showed spatial positive correlation and spatial agglomeration characteristics. Therefore, it is necessary to add spatial effects to the model for the study. Numerically, the *Moran’s I* index during the study period increased from 0.429 in 2005 to 0.474 in 2017, showing an overall upward trend, indicating that the significant spatial spillover characteristics presented by Chinese haze pollution are continuously strengthened over time. Figure 2 shows the *Moran’s I* index scatter plot of the distribution of local haze concentrations in China in 2005 and 2017. It shows that the local *Moran’s I* values of haze concentrations were mostly distributed in the first and third quadrants in 2005 and 2017, and that the spatial agglomeration was higher in 2017; this basically coincides with the spatial correlation and trend characteristics of haze pollution described in the social network analysis, indicating that the haze concentration in most areas of China is positively related to the surrounding areas.

### 5.2. Model Selection

The above *Moran’s I* test results show that there are spatial correlation characteristics of haze concentration among various provinces in China, so a measurement model with spatial effect is needed. First, model selection and testing were carried out for measurement analysis based on Stata14.0 software (from USA). The results are shown in Table 2 for unit root and consolidation tests of all data, which showed good stability of the panel data. Further, a Hausman test was performed, and the statistics were significant at the 1% level, thus rejecting the null hypothesis of random effects, and instead suggesting a fixed effect model. Secondly, the LM test showed that the statistics all passed the 5% significance test, so the non-spatial properties could be rejected, and the spatial measurement model was selected. Finally, when determining which model was more appropriate to choose based on LR and Wald tests, the correlation statistics were all significant at 1%, indicating that the spatial Durbin model cannot be reduced to a spatial lag model or spatial error model. In the SDM model, R^2^ and Log-L are larger than other models and can comprehensively reflect the spatial lag characteristics of explanatory and explained variables. Therefore, the SDM model can better reflect the dynamic game process of regulatory competition among provinces in China.

### 5.3. Empirical Results and Analysis

Table 3 shows the results of the heterogeneity of regulation strength and local government strategy selection based on the full sample data. First, the differential response coefficients $\rho_{1}$ and $\rho_{2}$ were significantly positive and both passed the significance level test of 1%. When environmental regulation in other regions is improved, local governments tend to imitate that behavior, and thereby enhance the regulation intensity. Further judging the relative size of $\rho_{1}$ and $\rho_{2}$, and the differentiated reaction coefficient $\rho_{1}$ > $\rho_{2}$, shows that there is “bottom-by-bottom competition” between Chinese provinces during the study period, that is, when the haze control efforts in neighboring areas are relaxed, the regional governments also tend to relax environmental regulation. The influence of the control variables was then examined. The coefficient of haze concentration is significantly positive, indicating that haze pollution is more serious when the local government uses stricter regulatory tools to achieve environmental performance. For the diffusion effect of haze, the local government reaction coefficient is not significant, which shows that when the government is selecting the haze coordination management strategy, the subjectivity of strict regulation is not strong. The coefficient of economic development level is significantly negative, indicating that although environmental inspectors have been included in the evaluation system, there is still a tendency to lower the regulatory threshold in order to attract investment in the coordinated control of haze. In terms of regional joint prevention and control, the improvement of economic development level and urbanization level in neighboring areas has had a positive effect on regulation, because the economic development of neighboring areas will attract the population out of the region and reduce the pressure of local environmental pollution to a certain extent. The coefficient of population density was positive, which matched the expected results. The growth of population brings about a sufficient rise of rigid energy needs, accompanied by many production activities, leading to the aggravation of haze pollution, thus forcing local governments to reduce pollution with stricter regulatory tools. The coefficient of energy strength is positive and is significant at the significance level of 1%, which shows that the decline in energy intensity causes a reduction in haze control intensity; that is, the increased efficiency of energy utilization (whether brought on by departmental labor force, capital investment, or technological innovation) can reduce air pollution and so reduce the pressure on local pollution control. The coefficient of urbanization level is positive, and similar findings were made by Li et al. [26]. At the current level of urbanization, the negative effect of air pollution exists objectively, the pressure of environmental public opinion and supervision of local governments has increased, and the environmental protection intensity has been further strengthened. The policy effect brought by the industrial structure is positive, which is consistent with the research results of Bo et al. [27]. It shows that the industrial structure of secondary industry is the pillar that leads to serious air pollution and promotes the implementation of the environmental protection supervision system.

Table 4 shows the analysis results of the heterogeneity of regulation intensity and local government strategy selection. Compared with the regression results of the whole sample, the reaction coefficients, $\rho_{1}$ and $\rho_{2}$, were significantly positive in different time periods, indicating that local governments imitated the choice of haze control strategies during the study period. To further judge the relative size of $\rho_{1}$ and $\rho_{2}$, consider that in 2005–2007, the reaction coefficient, $\rho_{1}$ > $\rho_{2}$, of regulation strength showed that there was “bottom-by-bottom competition” in the strategy choice of government haze collaborative governance. Although the government had put forward the binding indicators of pollutant discharge, hoping to promote a collaborative governance approach to controlling haze, some governments hoped to attract enterprises with high profits, and so sacrificing environmental regulations for economic benefits become their first choice. By contrast, the reaction coefficient of regulatory variables from 2008–2010 shows that the strategic choice of government haze coordination governance exhibits “top-by-top competition”, which reflects that after the environmental supervision system was officially incorporated into the assessment system, the competitive provinces with greater environmental governance had a greater influence on the strategy choice of local governments, resulting in stricter regulations and environmental supervision. Compared with the previous period, from 2011 to 2014 the ratio of the reaction coefficients, $\rho_{1}$ and $\rho_{2}$, of the local government regulatory variables decreased from 0.801 to 0.641; this shows that with the proposal of the “one-vote veto system” of the ecological assessment, the transformation of the environmental performance assessment mode forced enterprises to improve the enthusiasm of their collaborative haze management, and the regulation strength greatly improved. The response coefficient, $\rho_{1}$ < $\rho_{2}$, of the regulatory variables from 2015–2017 proved that when the intensity of haze control in competitive provinces during this period increased, the following response by local governments was significantly greater than their response when competitors relaxed. This reflects that with the improvement of the environmental protection performance assessment system, the demand for local environmental governance improved, and the subjective initiative of regional haze control was greatly stimulated.

The influences of the control variables over time were then examined. The coefficient of haze concentration on the regulation intensity is significantly positive, and the positive effect of haze pollution policies in the third and fourth stages is more obvious. This is because the State Council in 2013 issued the air pollution prevention action plan to the national prefecture level, and as a result urban inhalable particulate concentration fell by more than 10% as compared to 2012 standards; the plan also established a comprehensive detection network for haze prevention and control, and the severity of haze pollution inspired the government to further strengthen haze control regulations. The variables for economic development level were all negative except in the second stage. This shows that the proportion of economic development in the local government assessment system is too high, and that there is still ineffective supervision regarding environmental protection assessments. The population density variables were significantly positive over all four time periods. The increase in population density brings a regional industrial agglomeration effect, which makes the scale effect caused by haze control investment more significant, and more conducive to reducing haze pollution. Population agglomeration also increases the environmental public opinion and supervision pressure faced by the local government, thus forcing local governments to enhance environmental protection efforts. The energy structure coefficient is positive and increases significantly compared with the first phase value. In the early stage, there were more energy bases and heavy industrial bases, leading to a high proportion of high-emission energy varieties in the energy structure, and this “heavy” energy consumption structure is not easy to change in the short term. In the long run, the energy structure will have a positive impact on local regulation. The level of urbanization brought the positive effect of environmental regulation policies in the first and third stages, and the value continues to increase. With the advancement of urbanization, the increase in regional energy consumption, the continuous construction of infrastructure, and the increase in traffic congestion pressure will put greater pressure on the need for local haze control. The phased regression results of the industrial structure variables were not significant. This shows that the level of invisible environmental regulation dominated by secondary industry is low.

## 6. Conclusions and Recommendations

Competitive strategies for environmental control at the local government level emerge endlessly, but there is still little research on regional competition strategies for regional haze pollution control in China. This paper uses phased data of regional haze and analyzes the spatial spillover effect and association characteristics of various regions through social network analysis, and proves that the Beijing–Tianjin–Hebei region and the Yangtze River Delta region have always had the strongest haze correlation effect in China. In addition, the interprovincial data from 2005–2017 were used in a two-zone spatial Durbin model to analyze the serious phenomenon of “bottom-by-bottom competition” with respect to haze pollution prevention and control in China. In order to gain an economic advantage, local governments will relax the intensity of haze prevention and control with each other. The conclusion is robust, as each neighboring province presents a strong imitation effect toward it competitors. The conclusion of this paper suggests that regional collaborative environmental governance is directly related to the government’s performance assessment objectives, which should promote high quality economic development, human and natural destiny, and transformation of regional development model [28]. Based on the theoretical analysis and empirical results, some enlightenment can be proposed regarding how the central government can establish a coordinated strategy with the local government to control haze pollution.

The combination of the authoritative top-level design by the central government in various forms should be further strengthened to normalize the joint prevention and control of regional air pollution. To begin with, the establishment of effective coordinated control organizations has become an important part of air pollution control by local governments. In this regard, all regions should continue to deepen cooperation, especially with respect to the management of coordinated air pollution cooperation control work and solving possible conflicts in the governance process; at the same time, they should provide feasible policy suggestions regarding governance work, and should promote the bilateral cooperation development in the control of air pollution. Additionally, they should establish and improve cross-regional, bilateral, and multilateral cooperation governance rules. At present, the government mainly relies on the central government to set various environmental protection standards and assessment indicators (i.e., “political centralization”) while local governments at all levels are responsible for specific implementation (i.e., “governance centralization”). Centralized government at the implementation level can also have poor concerns, and this can lead to governance costs or opportunity costs. Therefore, establishing a system of cooperative governance rules can guarantee that the central policy is implemented, while maintaining local decision autonomy and initiative; even the regional governance theory of horizontal nested governance, i.e., the “river system”, and other territorial responsibility modes, such as urban agglomeration and metropolitan circle, can help build cooperation and collective unified rules, establishing multiple cooperation levels and other practical mechanism for collaboration [29].

## Figures and Tables

**Table 1:** Spatial correlation test for the core variables.

Year	*Moran’s I*	Z value
2005	0.429 ***	3.093
2006	0.452 ***	4.093
2007	0.456 ***	4.123
2008	0.419 ***	3.807
2009	0.373 ***	3.440
2010	0.396 ***	3.634
2011	0.468 ***	4.201
2012	0.397 ***	3.649
2013	0.474 ***	4.247
2014	0.402 ***	3.698
2015	0.447 ***	4.054
2016	0.476 ***	4.278
2017	0.474	4.263

Note: *** indicates *p* < 0.01.

**Table 2:** Model test results.

Variable	OLS	SAR	SEM	SDM
HC	−0.284 ***	−0.284 **	−0.342 **	−0.354 *
PG	0.154	0.076	0.115	−0.697 ***
PD	0.461 ***	0.423 ***	0.467 ***	0.504 ***
ES	−1.210 ***	−1.125 ***	−1.196 ***	−1.120 ***
URB	0.362	0.615	0.622	1.661 ***
IND	0.210	0.547 *	0.351	0.405 *
Adj R^2^	0.423	0.428	0.429	0.486
Log-like	——	−287.083	−287.980	−296.118
LM Spatial error	30.135 ***			
Robust LM-error	22.162 ***			
LM Spatial lag	18.720 ***			
Robust LM-lag	10.747 ***			

Note: *, **, *** indicates *p* < 0.10, *p* < 0.05, *p* < 0.01, respectively, with the same table below.

**Table 3:** Results of the two-zone model regression.

Variable	Coefficients	Z Value	*p* Value
HC	0.354 *	1.80	0.072
PG	−0.697 ***	−2.60	0.009
PD	0.504 ***	6.09	0.000
ES	1.120 ***	4.92	0.000
URB	1.661 ***	3.17	0.002
IND	0.405 *	1.68	0.093
W × HC	0.241	0.72	0.469
W × PG	0.929 ***	3.04	0.002
W × PD	−0.400 **	−2.54	0.011
W × ES	−0.399	−1.04	0.299
W × URB	−1.392 *	−1.77	0.076
W × IND	0.865 **	2.77	0.023
$\rho_{1}$	0.163 ***	2.70	0.007
$\rho_{2}$	0.151 ***	2.68	0.007
Adj R^2^	0.486		
Log-like	−286.118		

Note: *, **, *** indicates *p* < 0.10, *p* < 0.05, *p* < 0.01, respectively, with the same table below.

**Table 4:** Time-wise regression results.

Variable	2005–2007	2008–2010	2011–2014	2015–2017
HC	0.416 ***	0.352 *	0.419 **	0.579 **
	(2.17)	(1.85)	(2.25)	(2.01)
PG	−0.994 **	0.281	−1.607 ***	−0.430
	(−1.90)	(0.87)	(−3.01)	(−1.22)
PD	0.351 ***	0.344 ***	0.685 ***	0.341 ***
	(2.85)	(4.15)	(6.18)	(4.63)
ES	1.001 **	0.800 ***	0.822 **	0.258
	(2.57)	(2.55)	(2.35)	(1.37)
URB	1.796 ***	−0.488	4.729 ***	1.097
	(2.02)	(−0.79)	(4.77)	(1.36)
IND	0.637	−0.329	0.313	−0.070
	(1.17)	(−1.05)	(0.75)	(−0.27)
W × HC	0.789	0.103	−0.931 *	−0.03
	(1.46)	(0.26)	(−1.75)	(−0.09)
W × PG	0.454	1.590 ***	−1.138	0.934
	(0.68)	(2.94)	(1.57)	(1.41)
W × PD	−0.191	0.076	−0.430 **	−0.303 *
	(−0.86)	(0.45)	(−1.98)	(−1.92)
W × ES	−1.036	−0.894	−0.626	−0.412
	(−1.58)	(−1.50)	(−1.00)	(−1.34)
W × URB	−0.835	−1.793 *	1.310	−2.426
	(−0.65)	(−1.66)	(0.82)	(−1.54)
W × IND	1.859 *	0.240	2.170 **	−0.303
	(1.73)	(0.35)	(2.36)	(−0.62)
$\rho_{1}$	0.162 *	0.115 ***	0.075 ***	0.160 *
	(1.55)	(4.87)	(3.46)	(1.79)
$\rho_{2}$	0.158 **	0.142 ***	0.117 *	0.170 *
	(2.95)	(2.72)	(5.99)	(1.96)
Adj R2	0.382	0.780	0.815	0.734
Log-like	−57.437	−25.310	−44.208	−40.878

Note: *, **, *** indicates *p* < 0.10, *p* < 0.05, *p* < 0.01, respectively, with the same table below.

## Data Availability

All data included in this study are available upon request by contact with the corresponding author.
